# Comprehensive analysis of miRNA–mRNA interactions in ovaries of aged mice

**DOI:** 10.1111/asj.13721

**Published:** 2022-04-13

**Authors:** Jihyun Kim, Sooseong You

**Affiliations:** ^1^ Clinical Medicine Division Korea Institute of Oriental Medicine Daejeon South Korea

**Keywords:** differentially expressed genes, functional annotation, microRNAs, oocyte, ovarian aging

## Abstract

Advanced maternal age and ovarian aging are deleterious to the quantity and quality of oocytes and epigenetic modifications, which can affect the health of offspring. However, relatively little is known about the regulation of microRNA‐mediated transcription during ovarian aging. We therefore aimed to identify age‐related mRNA and microRNA changes and their interactions in the ovaries of aged mice. We performed QuantSeq 3′mRNA and small RNA sequencing to compare their expression patterns in post‐ovulation ovaries from young (12‐week‐old) and old (44‐week‐old) mice. Functional annotation and integrative analyses were performed to identify the potential functions of differentially expressed genes and identify binding sites for critical microRNAs. We found 343 differentially expressed genes and 9 microRNAs in our comparison of the two mouse groups, with fold changes >2.0 (*P* < 0.01). Furthermore, we identified possible direct interactions between 24 differentially expressed mRNAs and 8 microRNAs. The differentially expressed genes are involved in fat digestion and absorption, the PI3K‐Akt signaling pathway, serotonergic synapse, and ovarian steroidogenesis, which are important for folliculogenesis and oocyte growth. During ovarian aging, changes in gene expression induce alterations in folliculogenesis, oocyte growth, and steroidogenesis, resulting in decreased oocyte quality and reproductive outcomes.

## 1 INTRODUCTION

The improvement in human longevity has been accompanied by a marked increase in aging‐related problems. Age‐linked changes, including homogenomic instability, epigenetic alterations, mitochondrial dysfunction, and cellular senescence, occur over the course of the aging process (Rebelo‐Marques et al., 2018). In mammals, a fixed pool of primordial follicles in the ovaries serves as a source of developing follicles and subsequently oocytes during the entire reproductive cycle of the organism (Wang et al., 2017). The gradual decline in fertility results from ovarian aging, which is characterized by a reduction in the quantity and quality of ovarian follicles (Zhang, Chen, et al., 2019). The ovarian aging process leads to an increased rate of aneuploidy in early embryos (Broekmans et al., 2009). In particular, women with advanced maternal age, particularly those over 40 years old, also have an increased risk of chromosomal abnormalities and epigenetic aberrations in oocytes (Cleary‐Goldman et al., 2005; Ge et al., 2015; Laopaiboon et al., 2014).

During the last decade, messenger RNA (mRNA) expression profiling has been widely used to investigate changes in gene expression in aging ovaries. Many systematic approaches have revealed that gene regulation by epigenetic reprogramming occurs during folliculogenesis, oogenesis, and early embryogenesis (Bromfield et al., 2008; Zhang & Smith, 2015). Mammalian microRNAs (miRNAs) are small non‐coding RNAs that regulate the post‐transcriptional expression of target genes. miRNA‐mediated post‐transcriptional regulation plays important roles during many aspects of mammalian reproduction, ranging from the maturation of germ cells to the initiation of gastrulation (Kim et al., 2019; Reza et al., 2019). In addition, several studies have examined the expression of miRNAs in aging organs of mice and humans (Gonzalo, 2010). Elucidating the molecular events underlying the aging process in ovaries is important for developing approaches to prevent female reproductive problems associated with age. However, little is known about how miRNA‐mediated transcription is regulated during ovarian aging.

In this study, we aimed to identify age‐related mRNA and miRNA changes, and their interactions, in the ovaries of aged mice.

## 2 MATERIALS AND METHODS

### 2.1 Mice

All experiments and analyses were conducted in accordance with the ARRIVE guidelines and regulations. The animal experimental protocols were approved by the Institutional Animal Care and Use Committee of the Korea Institute of Oriental Medicine, Daejeon, Korea (approval number 20‐090). Female BALB/c mice aged 12 and 44 weeks (Central Lab Animal Inc., Seoul, Korea) were housed under specific pathogen‐free conditions.

The mice were treated with 5 IU of pregnant mare serum gonadotropin (Prospec, Rehovot, Israel) and 5 IU of human chorionic gonadotropin (hCG; Prospec) to induce superovulation of oocytes for assessment. Hormonally stimulated ovaries were removed post‐ovulation and immediately placed in liquid nitrogen, prior to processing for mRNA and small RNA sequencing. RNA profiling was performed using gonadotropin‐stimulated ovaries to analyze mRNAs and miRNAs contributing to the clinical phenotypes of poor oocyte quantity and quality.

### 2.2 Assessment of oocyte quantity and quality

Oocytes were collected 18 h post‐hCG injection in preincubated human tubal fluid medium (Irvine Scientific, Santa Ana, CA, USA), fixed with 4% paraformaldehyde (Biosesang), permeabilized with 0.5% Triton X‐100 (Sigma‐Aldrich, St. Louis, MO, USA) for 10 min, and blocked with phosphate‐buffered saline containing 3% bovine serum albumin (GenDEPOT, Katy, TX, USA). Thereafter, the oocytes were incubated with a rabbit anti‐α‐tubulin antibody (1:200; Cell Signaling Technology, Danvers, MA, USA) and subsequently mounted on slides using VECTASHIELD antifade mounting medium with 4,6‐diamidino‐2‐phenylindole (Vector Laboratories, Peterborough, UK) to visualize the chromosomes using a fluorescence microscope (BX51; Olympus, Tokyo, Japan). Oocytes with well‐organized bipolar spindles and tightly aligned chromosomes at metaphase were scored as normal. Oocytes with dispersed chromosomes or spindle disassembly were scored as abnormal.

### 2.3 RNA sequencing for mRNA and small RNA expression

Hormonally stimulated ovaries were collected from the mice post‐ovulation, and total RNA was extracted using TRIzol (Invitrogen, Carlsbad, CA, USA) according to the manufacturer's instructions. The purity and integrity of the extracted RNA were evaluated using a NanoDrop ND‐2000 spectrophotometer (Thermo Fisher Scientific, Waltham, MA, USA) and Agilent 2100 bioanalyzer (Agilent Technologies, Amstelveen, The Netherlands). All samples showed high purity (optical density [OD]_260_/OD_280_ > 1.80) and integrity (RNA integrity number > 7.0). Sequencing was performed using the Illumina NextSeq 500 platform following the vendor's instruction by E‐biogen, Inc. (Seoul, Korea). A fold‐change value of >2.0 and a *p* value of <0.01 were used as thresholds to identify differentially expressed genes.

### 2.4 Integrative analysis of mRNA and microRNA expression profiles

Gene enrichment and functional annotation analysis for a significant probe list were performed using Gene ontology (GO) (http://geneontology.org) and the Kyoto Encyclopedia of Genes and Genomes (KEGG; http://kegg.jp) to identify the potential functions of differentially expressed genes (DEGs) in biological processes. Statistical significance threshold of enrichment analysis was set at *p* < 0.05. The DEGs associated with four KEGG pathways were introduced into STRING (http://string-db.org, version 11.0) to build the protein–protein interaction network interaction (maximum number of interactors = 0 and confidence score > 0.4) (The UniProt Consortium, 2017).

mRNAs and miRNAs that had been differently expressed between young and old mice with significance (*p* < 0.05) based on sequencing were included in the analysis. TargetScan 7.2 webtool (www.targetscan.org, accessed March 2018) was used to identify binding sites of critical miRNAs and DEGs based on a context++ model of miRNA targeting efficacy (Agarwal et al., 2015).

### 2.5 Statistical analysis

Data are presented as mean ± SEM. The statistical significance of differences between two groups was determined by a Student's *t* test using GraphPad Prism version 8.4.0 (GraphPad Software, Inc., La Jolla, CA, USA). Differences were considered statistically significant at *p* < 0.05.

## 3 RESULTS

### 3.1 Decline in oocyte quantity and quality with aging

**FIGURE 1:**
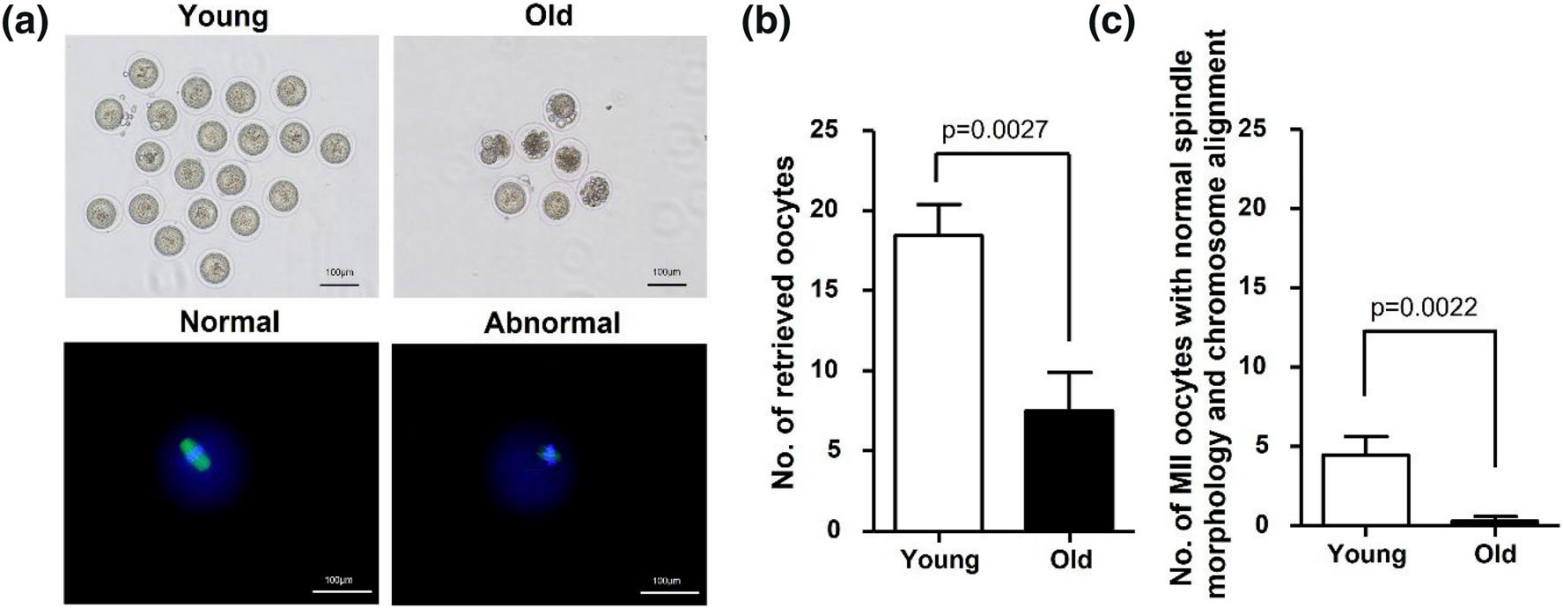
Quantity and quality of mouse oocytes. Oocytes retrieved from young (*n* = 10) and old (*n* = 10) mice at 18 h after hCG injection. (a) Representative images of retrieved oocytes, as observed under the microscope (blue fluorescence: chromosome; green fluorescence: spindle). Number of retrieved oocytes (b) and MII oocytes with normal spindle morphology and chromosomal alignment (c) from young and old mice. Data are presented as mean ± standard error of the mean. Statistical analysis was performed using the Students *t* test. Young: 12‐week‐old mice; old: 44‐week‐old mice

To assess, the quantity and quality of oocytes under conditions of aging, young, and old mice were hormonally induced to undergo superovulation. Retrieved oocytes were observed under the microscope, and their quantity and quality were assessed (Figure 1a). The numbers of total retrieved oocytes and mature metaphase II (MII) oocytes with normal chromosomes and well‐organized spindle alignments were significantly lower in old mice than in young mice (Figure 1b,c). These results indicate that aging is a risk factor for poor oocyte quality and quality.

### 3.2 Age‐related mRNA expression in mouse ovaries

**FIGURE 2:**
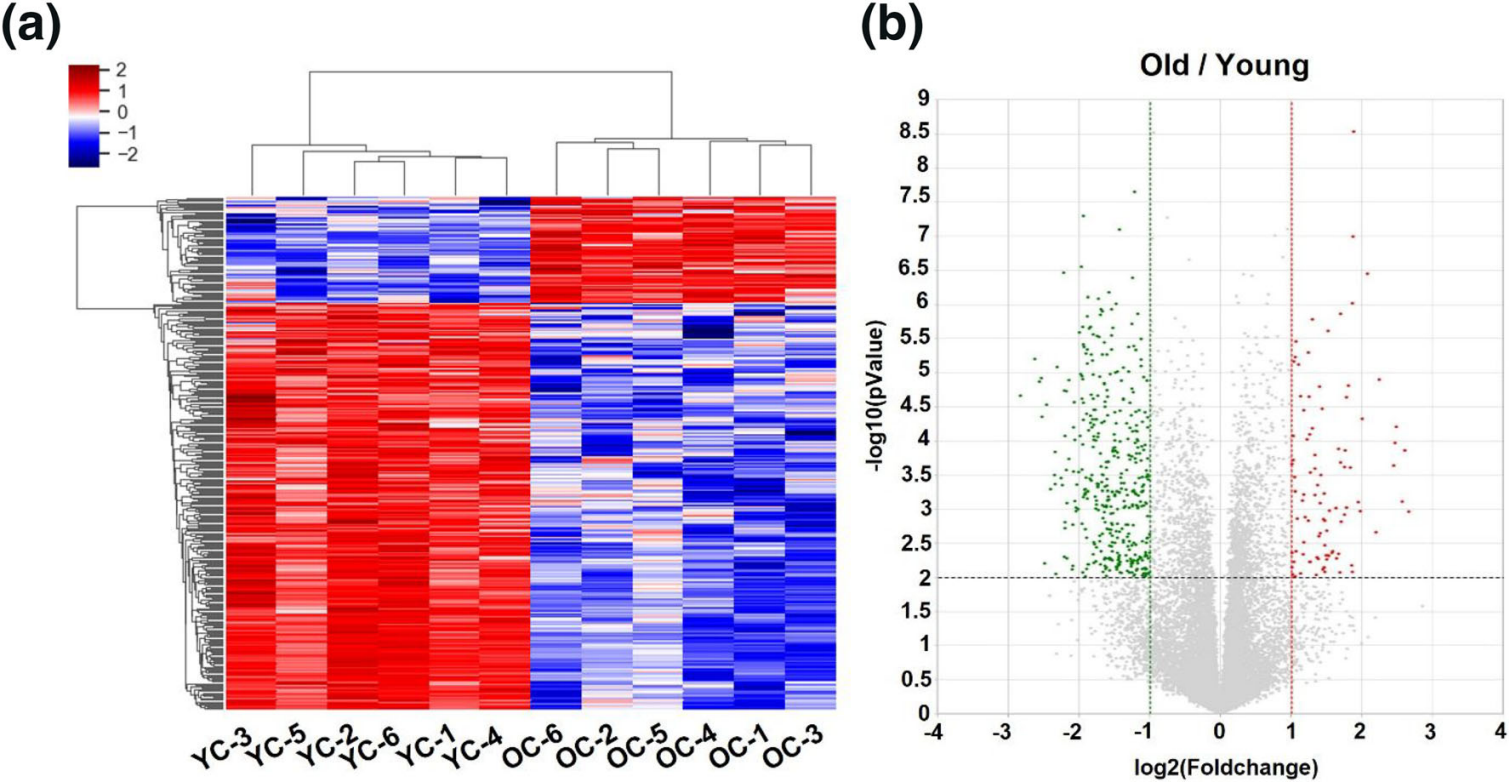
Hierarchical clustering and analysis of differentially expressed mRNAs. QuantSeq 3mRNA analysis was performed to compare gene expression in post‐ovulation ovaries from young (*n* = 6) and old (*n* = 6) mice. (a) Hierarchical clustering analysis showed that 343 genes displayed significantly different levels of expression in young and old mouse groups. (b) Volcano plot of 343 differentially expressed mRNAs between young and old mice, with a fold‐change > 2.0 and *p* < 0.01. Red and green dots indicate upregulated and downregulated differentially expressed mRNAs, respectively. Young: 12‐week‐old mice; old: 44‐week‐old mice

We next performed QuantSeq 3 mRNA sequencing to compare the mRNA expression patterns in post‐ovulation ovaries from young and old mice. Sequencing identified 3,878 genes, and hierarchical clustering analysis revealed 343 DEGs that exhibited different expression patterns between young and old mice (Figure 2a), and a volcano plot revealed these 343 DEGs to have fold changes > 2.0 (*p* < 0.01) (Figure 2b). Bioinformatics analysis was performed on 71 upregulated genes (20.7%) and 272 downregulated genes (79.3%), as shown in Tables S1 and S2.

**FIGURE 3:**
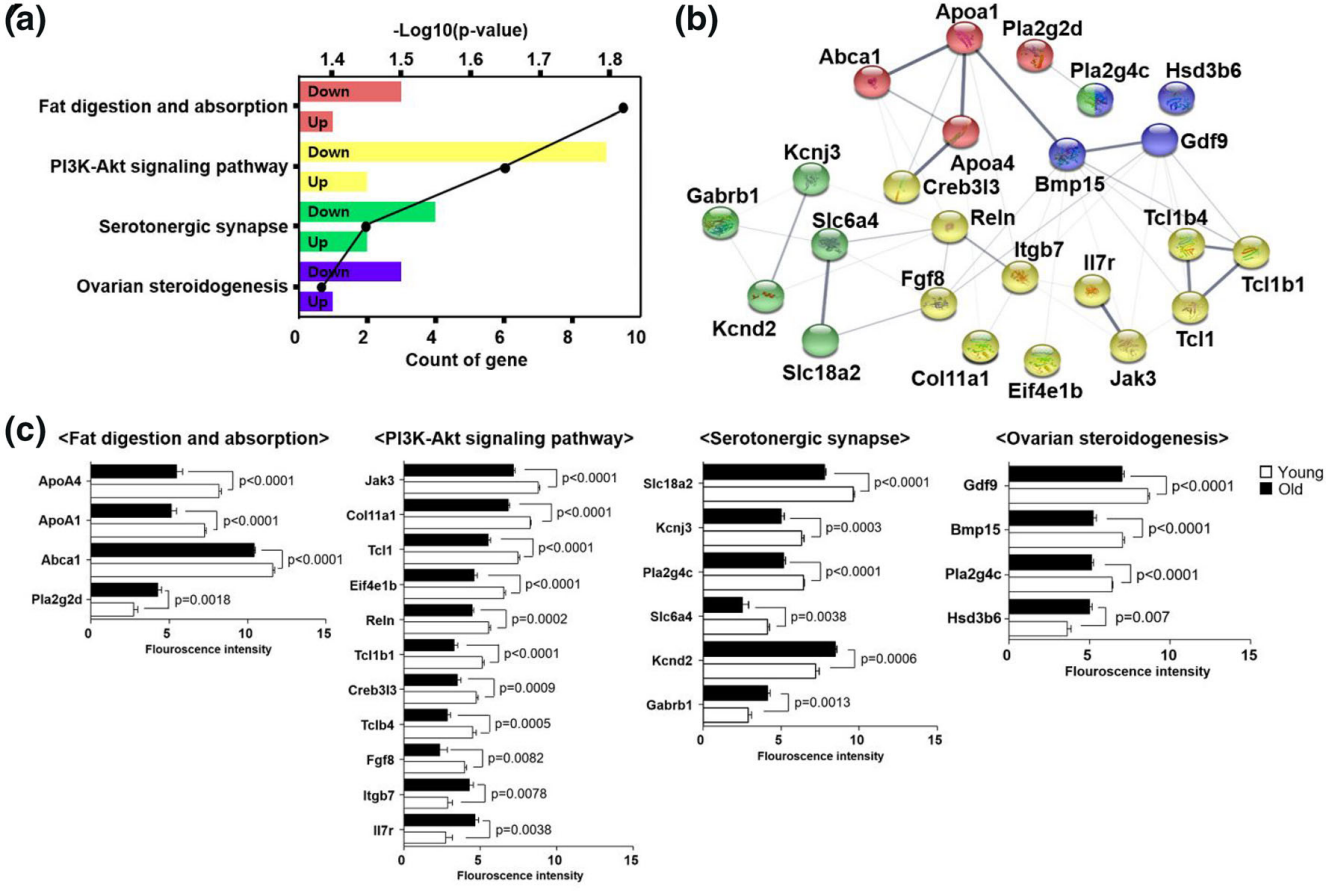
Functional annotation of differentially expressed genes. Kyoto encyclopedia of genes and genomes (KEGG) analysis was performed to identify potential signaling pathways enriched with differentially expressed genes. (a) Differentially expressed genes are involved in fat digestion and absorption, PI3K‐Akt signaling, serotonergic synapse, and ovarian steroidogenesis. Up: upregulated genes in old mice compared to young mice; down: downregulated genes in old mice compared to young mice. (b) A protein–protein interaction network of these genes was predicted using STRING (http://string‐db.org, version 11.0). (c) The expression of genes associated with the four KEGG pathways was compared between young and old mice. Data are presented as mean ± standard error of the mean. Statistical analysis was performed using the Students *t* test. Young: 12‐week‐old mice; old: 44‐week‐old mice

GO analysis of the young and old mouse datasets revealed that the DEGs were enriched in biological processes, such as positive regulation of cell proliferation and fatty acid biosynthetic process, ovarian follicle development, meiotic cell cycle, cell adhesion, and oogenesis (Table 1). The KEGG pathway analysis revealed that the DEGs were involved in fat digestion and absorption, phosphatidylinositol 3‐kinase/protein kinase B (PI3K‐Akt) signaling pathway, serotonergic synapse, and ovarian steroidogenesis (Figure 3a). We also constructed a network of DEGs using the STRING tool (Figure 3b). Among 24 DEGs enriched in four pathways, six had significantly higher expression levels while 18 had significantly lower expression levels in old than in young mice (Figure 3c).

**TABLE 1:** Top 10 biological processes associated with differentially expressed mRNAs in the ovaries of young and old mice

Biological process		*p* value	Genes
GO:0045596	Negative regulation of cell differentiation	<0.001	GM13023, GM13103, OOG4, IHH, OOG3, GM13084, OOG1, C87499, C87977, PRAMEF12, C87414, GM10436, GM2042
GO:0045723	Positive regulation of fatty acid biosynthetic process	<0.001	SLC45A3, RGN, APOA1, APOA4, AGT
GO:0008284	Positive regulation of cell proliferation	<0.001	GM13023, NTRK2, MYOCD, GM13103, OOG4, IHH, OOG3, GM13084, AGT, OOG1, GDF9, C87499, ADCYAP1, ESM 1, FGF8, FABP4, C87977, PRAMEF12, C87414, GM10436, GM2042
GO:0001541	Ovarian follicle development	<0.001	ADCYAP1, PCYT1B, SOHLH1, NOBOX, OAS1D, ESR2, BMP15
GO:0030154	Cell differentiation	<0.001	PTPRU, DDX4, ARHGEF28, LECT1, IHH, TEX19.1, DMKN, ELAVL3, ADGRG1, FGF8, SOHLH1, NTNG1, NTRK2, BATF3, STYK1, TEX15, PLET1, TDRD5, KAZALD1, TDRD1, DAZL, NLRP14, BMP3, NOBOX, BMPR1B, GTSF1
GO:0051321	Meiotic cell cycle	<0.001	H1FOO, TDRD1, DDX4, WEE2, MNS1, TEX15, TEX19.1, SMC1B
GO:0051607	Defense response to virus	<0.001	OAS1H, IL33, RSAD2, H2‐Q9, OAS1A, OAS1C, OAS1D, OAS1E, IFIT1BL2, IFIT3
GO:0007155	Cell adhesion	<0.001	MYBPC3, PTPRU, NRXN1, OMD, DPT, IZUMO1R, MSLN, BCAN, ACAN, ADGRG1, NFASC, RELN, ASTL, GPNMB, NINJ2, PERP, ITGB7, RADIL
GO:0042157	Lipoprotein metabolic process	0.0012	ABCA1, APOL9B, APOC3, APOA1, APOA4
GO:0048477	Oogenesis	0.0015	DAZL, SOHLH1, NOBOX, YBX2, FMN2

*Note*: Targets were subjected to pathway analysis and subsequently classified based on their enrichment in biological processes. Young: 12‐week‐old mice; old: 44‐week‐old mice.

### 3.3 Age‐related miRNA expression in mouse ovaries

**FIGURE 4:**
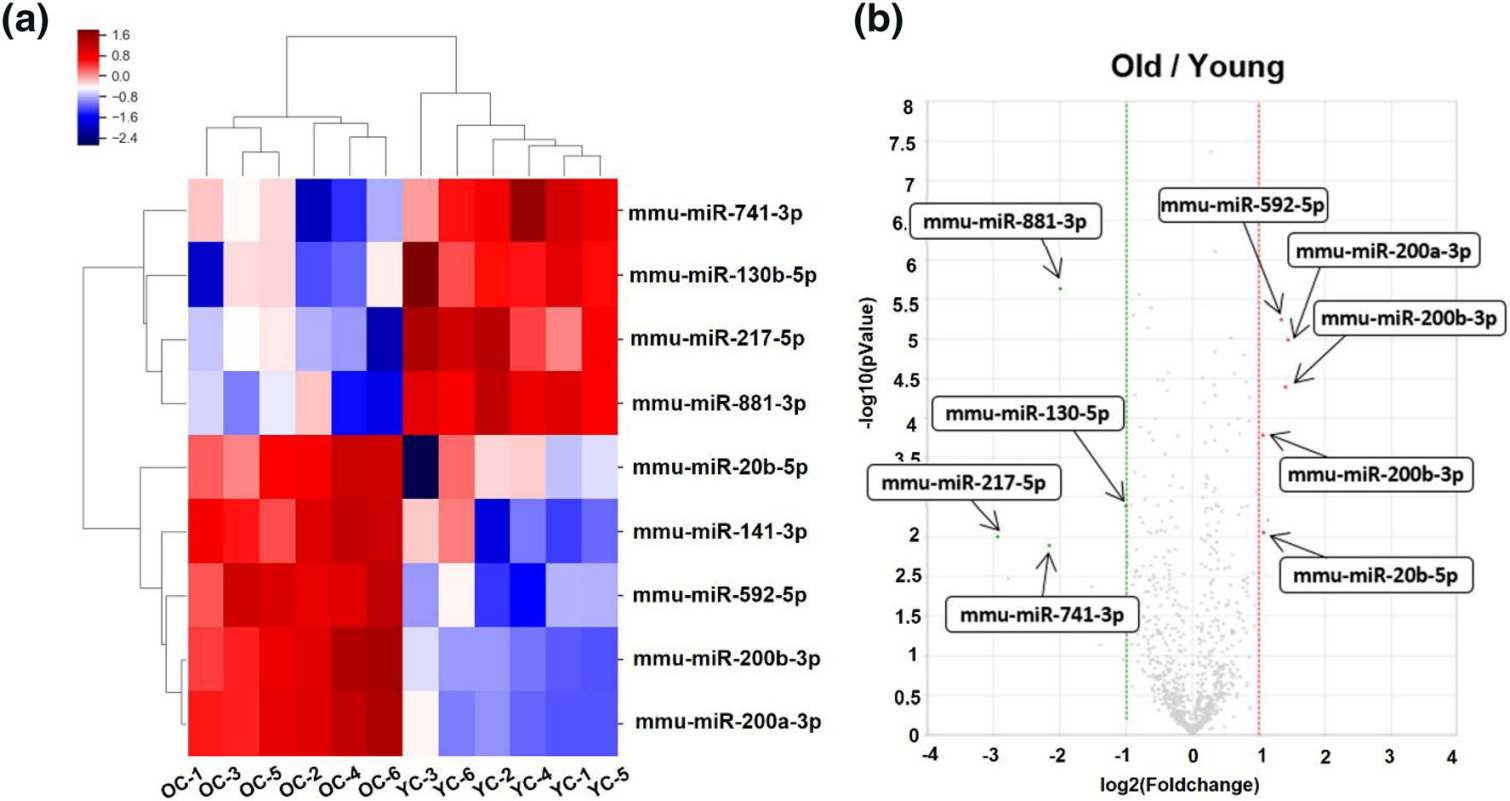
Hierarchical clustering and analysis of microRNAs. Quantseq small RNA analysis was performed to compare gene expression in post‐ovulation ovaries from young (*n* = 6) and old (*n* = 6) mice. (a) Hierarchical clustering analysis showed that nine miRNAs displayed significantly different levels of expression between young and old mouse groups. (b) Volcano plot of nine differentially expressed miRNAs between young and old mice, with a fold‐change > 2.0 and *p* < 0.01. Young: 12‐week‐old mice; old: 44‐week‐old mice

We performed small RNA sequencing to compare the miRNA expression patterns in post‐ovulation ovaries from young and old mice. Small RNA sequencing identified 175 miRNAs, and hierarchical clustering analysis showed that nine miRNAs displayed significantly different levels of expression in young and old mice (Figure 4a), and a volcano plot showed that these nine miRNAs had fold changes > 2.0 (*p* < 0.01) (Figure 4b). Of these miRNAs, five were upregulated (55.6%), and four were downregulated (44.4%) (Table 2).

**TABLE 2:** List of microRNAs altered in old compared to young mice

	Gene symbol	Fold change	*p* value
Upregulated	Mmu‐miR‐141‐3p	2.096	<0.001
	Mmu‐miR‐20b‐5p	2.111	0.003
	Mmu‐miR‐592‐5p	2.547	<0.001
	Mmu‐miR‐200b‐3p	2.657	<0.001
	Mmu‐miR‐200a‐3p	2.729	<0.001
Downregulated	Mmu‐miR‐217‐5p	0.130	0.003
	Mmu‐miR‐741‐3p	0.223	0.004
	Mmu‐miR‐881‐3p	0.252	<0.001
	Mmu‐miR‐130b‐5p	0.499	0.001

*Note*: Comparison of expression data in young and old mice revealed nine differentially expressed miRNAs with fold changes > 2.0, and *p* < 0.01. Young: 12‐week‐old mice; old: 44‐week‐old mice.

### 3.4 Integrative analysis of differentially expressed mRNAs and miRNAs

Further enrichment analysis using the TargetScan webtool identified possible direct interactions between differentially expressed mRNAs and miRNAs. We found that 24 DEGs harbored binding sites for the eight miRNAs, as summarized in Tables 3 and 4. Of note, the majority of interacting miRNAs, including miR‐200b‐3p, miR‐741‐3p, miR‐200a‐3p, and miR‐141‐3p, harbored binding sites for DEGs involved in folliculogenesis and steroidogenesis.

**TABLE 3:** Binding sites between upregulated mRNAs and microRNAs

Target	Position	miRNA	5′ (mRNA)	3′ (miRNAs)
Adcyap1	1,262–1,268	miR‐130b‐3p	UCCACAGAGAAAUUA**UGCACUA**A	UACGGGAAAGUAGUA**ACGUGA**C
Fabp4	386–392	miR‐881‐3p	CUUGGGUAAUCCUAG**ACACAGU**G	AGAUAAGUCUUUUC**UGUGUCA**A
Itgb7	621–628	miR‐20b‐5p	GAAGGAACAUACACU‐‐‐‐**GCACUUUA**	GAUGGACGUGAUACU**CGUGAAA**C
Radil	362–368	miR‐200a‐3p	AACACUGAAGGAAGC**CAGUGUU**G	UGUAGCAAUGGUCU**GUCACAA**U
	362–368	miR‐141‐3p	AACACUGAAGGAAGC**CAGUGUU**G	GGUAGAAAUGGUCU**GUCACAA**U

*Note*: Bold text indicates the binding sites predicted by TargetScan analysis (www.targetscan.org, accessed March 2018).

**TABLE 4:** Binding sites between downregulated mRNAs and microRNAs

Target	Position	miRNA	5′ (mRNA)	3′ (miRNAs)
Agt	2,240–2,247	miR‐592‐5p	CAGCUGUGUCAAGUU**GACACAAA**	UGUAGUAGCGUAUAA**CUGUGUU**A
	369–375	miR‐200b‐3p	GUAAUUAGCUCACUG**AGUAUUA**G	AGUAGUAAUGGUCCG‐‐**UCAUAAU**
	489–496	miR‐200b‐3p	GUGAUAAGCUAAACA**CAGUAUUA**	AGUAGUAAUGGUCC**GUCAUAA**U
	1,242–1,248	miR‐741‐3p	CUCUGAGGCCCGGCU**UCUCUCA**U	AGAUGUAUCUUACCGU**AGAGAG**U
	486–493	miR‐881‐3p	UCAGUGAUAAGCUAA**ACACAGUA**	AGAUAAGUCUUUUC**UGUGUCA**A
Astl	286–292	miR‐200b‐3p	UCAAGAGCAAGGUUC**CAGUAUU**G	AGUAGUAAUGGUCC**GUCAUAA**U
Arhgef28	46–52	miR‐20b‐5p	AAACAUAAACCACUG‐‐‐**GCACUUU**G	GAUGGACGUGAUACU**CGUGAAA**C
Bmp15	1,216–1,222	miR‐200b‐3p	UACUGUUCUUCUCUU**CAGUAUU**C	AGUAGUAAUGGUCC**GUCAUAA**U
	1,298–1,304	miR‐200b‐3p	CCCUUAAAAUGCUCU**AGUAUUA**C	AGUAGUAAUGGUCCG**UCAUAA**U
	246–253	miR‐741‐3p	UUAAGCAUUGUUUAA**AUCUCUCA**	AGAUGUACUUACCG**UAGAGAG**U
C87499	696–703	miR‐200b‐3p	AUUAUUGUGGUUUGA**CAGUAUUA**	AGUAGUAAUGGUCC**GUCAUAA**U
	203–209	miR‐741‐3p	UUCUAUGAAAAUACC**AUCUCUC**U	AGUGUAUCUUACCG‐‐**UAGAGAG**U
Dazl	1744–1751	miR‐130b‐3p	AUUUAAGAGAAGGGA**GAAAGAGA**	UCAUCACGUUGUCC**CUUUCUC**A
Esr2	1882–1889	miR‐200a‐3p	UGGAAACUAUUAGUU‐‐**CAGUGUUA**	UGUAGCAAUGGUCU**GUCACAA**U
	1882–1889	miR‐141‐3p	UGGAAACUAUUAGUU‐‐**CAGUGUUA**	GGUAGAAUGGUCU**GUCACAA**U
Fgf8	166–172	miR‐130b‐3p	UUUGUUUUUUAAACA**AAAGAGA**G	UCAUCACGUUGUCCC**UUUCUC**A
Mns1	354–360	miR‐741‐3p	AAACAGCUAUUUACU**UCUCUCA**A	AGAUGUAUCUUACCGU**AGAGAG**U
Nrxn1	286–292	miR‐141‐3p	AUUCCCUAACAUCCG**CAGUGUU**U	GGUAGAAAUGGUCU**GUCACAA**U
Ninj2	178–184	miR‐20b‐5p	CUGGGUGACGUAAUU**GCACUUU**G	GAUGGACGUGAUACU**CGUGAAA**C
Oog4	26–32	miR‐200b‐3p	AAGAAAUGGAAGCUG**AGUAUUA**G	AGUAGUAAUGGUCCG**UCAUAA**U
Oas1d	206–212	miR‐141‐3p	UGCCUUAGCUUCCAA**CAGUGUU**C	GGUAGAAAUGGUCU**GUCACAA**U
	421–427	miR‐141‐3p	CCCUGGGAAUCUGGC**CAGUGUU**C	GGUAGAAAUGGUCU**GUCACAA**U
	206–212	miR‐200a‐3p	UGCCUUAGCUUCCAA**CAGUGUU**C	UGUAGCAAUGGUCU**GUCACAA**U
	421–427	miR‐200a‐3p	CCCUGGGAAUCUGGC**CAGUGUU**C	UGUAGCAAUGGUCU**GUCACAA**U
Pcyt1b	1,061–1,067	miR‐20b‐5p	UCCCUCAUGACUUGG‐**GCACUUU**G	GAUGGACGUGAUACU**CGUGAAA**C
Pramef12	535–541	miR‐130b‐3p	CACUGAGAGGAAUGC**AAAGAGA**G	UCAUCACGUUGUCCC**UUUCUC**A
	902–908	miR‐200a‐3p	AUGUAGGGGAAUUUU**CAGUGUU**C	UGUAGCAAUGGUCU**GUCACAA**U
	902–908	miR‐141‐3p	AUGUAGGGGAAUUUU**CAGUGUU**C	GGUAGAAAUGGUCU**GUCACAA**U
Pla2g4c	420–426	miR‐741‐3p	AGCCUCAUAAAUUUU**UCUCUCA**U	AGAUGUAUCUUACCGU**AGAGAG**U
	1,089–1,096	miR‐741‐3p	AUUUUUCCUUUUUUU**AUCUCUC**A	AGAUGUAUCUUACCG**UAGAGAG**U
Reln	252–258	miR‐200b‐3p	UAUCAGUUACAGUGG**CAGUAUU**G	AGUAGUAAUGGUCC‐**GUCAUAA**U
	341–347	miR‐200b‐3p	AGUGGCAUUUUAGCA**CAGUAUU**U	AGUAGUAAUGGUCC**GUCAUAA**U
Slc6a4	367–373	miR‐141‐3p	UGCUUCUAAAGCCUU**CAGUGUU**C	GGUAGAAAUGGUCU**GUCACAA**U
	367–373	miR‐200a‐3p	UGCUUCUAAAGCCUU**CAGUGUU**C	UGUAGCAAUGGUCU**GUCACAA**U
Tdrd1	33–40	miR‐130b‐3p	AAUAAACACUGGGAA**GAAAGAGA**	UCAUCACGUUGUCC**CUUUCUC**A
	945–951	miR‐592‐5p	UCACUGUCUUCUCAA**ACACAAA**U	UGUAGUAGCGUAUAAC**UGUGUU**A
	777–783	miR‐741‐3p	AGGAUGUGCACUGCU**UCUCUGA**G	AGAUGUAUCUUACCGU**AGAGAG**U
	1,061–1,067	miR‐741‐3p	CUGACAGCCCAGUAG**UCUCUCA**U	AGAUGUAUCUUACCGU**AGAGAG**U
Tex19.1	176–182	miR‐200a‐3p	CUGGCAUGUUCGUGU**CAGUGUU**C	UGUAGCAAUGGUCU**GUCACAA**U
	183–189	miR‐200a‐3p	CUGGCAUGUUCGUGU**CAGUGUU**C	GGUAGAAAUGGUCU**GUCACAA**U
	176–182	miR‐141‐3p	GUUCGUGUCAGUGUU**CAGUGUU**U	UGUAGCAAUGGUCU**GUCACAA**U
	183–189	miR‐141‐3p	GUUCGUGUCAGUGUU**CAGUGUU**U	GGUAGAAAUGGUCU**GUCACAA**U

*Note*: Bold text indicates the binding sites predicted by TargetScan analysis (www.targetscan.org, accessed March 2018).

## 4 DISCUSSION

Advanced maternal age has deleterious effects on the quantity and quality of oocytes and epigenetic modifications, and it affects the health of the offspring (Takeo et al., 2013). Changes in epigenetic modifications, including DNA methylation, histone, and RNA modification, can cause several aging‐related diseases, such as degenerative diseases and infertility (Pagiatakis et al., 2019). Networks of miRNAs affect the molecular mechanisms that control aging during the life span of an organism (Liang et al., 2009). miRNAs have recently been explored to treat steroid‐related disorders and female infertility (Virant‐Klun et al., 2016). To investigate the transcriptomic changes, our study used ovaries from 12 and 44 weeks old mice corresponding to 20 and 40 years of women, respectively (te Velde & Pearson, 2002; Wang et al., 2020).

Using RNA sequencing, we identified important genes and pathways involved in maintaining the ovarian function. Many DEGs were involved in lipid‐related metabolic pathways, including fat digestion and absorption. Metabolic profiling has revealed that aging alters the composition of follicular fluid, which includes many lipids and small metabolites that may be associated with oocyte competence (O'Gorman et al., 2013). Phospholipase A2, group IID, is an arachidonic acid, and its metabolites play important roles in the metabolic and endocrine functions of ovarian and placental cells (Boone et al., 1993; Cordeiro et al., 2018). Women with polycystic ovarian syndrome exhibited lower levels of apolipoprotein A1 than did normal women (Couto Alves et al., 2017). In addition, arachidonic acid serves as a cell‐signaling intermediate responsible for modulating cyclic AMP activation and PI3K/Akt, which regulate ovarian cellular growth and proliferation (Hughes‐Fulford et al., 2006; Zhang, Wang, et al., 2019). The PI3K/Akt signaling pathway plays important roles in follicular growth and atresia. High PI3K/Akt activity is linked to a decline in the number of primordial follicles and ovarian aging due to the regulation of follicular recruitment by this pathway. In contrast, inhibition of PI3K/Akt accelerates apoptosis of granulosa cells and leads to premature ovarian failure (Zheng et al., 2012). Spatiotemporal control of PI3K/Akt activation and function may contribute to the maintenance of the primordial follicle pool and oocyte maturation.

Ovarian aging alters not only ovarian follicular activation but also steroidogenesis. As they age, women produce lower levels of estradiol and other estrogen hormones (Gosden & Faddy, 1994). Dysregulation of feedback in the pituitary gland to regulate the level of follicle‐stimulating hormone affects ovarian follicular development and compromises the chances of pregnancy (Stilley & Segaloff, 2018). The expression of growth differentiation factor 9 (*Gdf9*) and bone morphogenetic protein 15 (*Bmp15*) can serve as a predictor of ovarian aging. Age‐related decline in the expression of these two genes is associated with poor ovarian response to ovarian hyperstimulation, resulting in poor oocyte quantity and quality (Gong et al., 2021). Interestingly, our analyses revealed that *Bmp 15* harbors two binding sites for miR‐200b‐3p and one for miR‐741‐3p. However, functional analysis of the interaction between them is necessary. Our RNA sequencing analyses predicted that altered gene expression influences ovarian aging, leading to decreased oocyte quality.

Furthermore, GO analysis revealed several genes involved in cell differentiation, proliferation, and the meiotic cell cycle. Both 2′‐5′ oligoadenylate synthetase 1D (*Oas1d*) and estrogen receptor 2 (*Esr2*), which are known to be involved in folliculogenesis and oocyte growth, harbor binding sites for miR‐141‐3p and miR‐200a‐3p. Decreased expression of *Oas1d* has been shown in mouse oocytes exposed to cyclophosphamide which induces oxidative damage to DNA structural breaks and mutations (Kim & You, 2020; Spears et al., 2019). This interaction between these two mRNAs and two miRNAs may contribute to the maintenance of normal ovarian function and may be deregulated during the aging process. Angiotensinogen (*Agt*), one of the identified DEGs that interacts with miR‐200b‐3p, miR‐741‐3p, and miR‐881‐3p, plays a role in the local renin‐angiotensin system in the ovary. *Agt* has been shown to participate in folliculogenesis, steroidogenesis, oocyte maturation, and ovulation (Reis et al., 2011; Yoshimura, 1997).

There are three limitations to our current study. First, we were unable to perform functional validations of our findings. Functional analysis of DEGs will be necessary to develop strategies or approaches for preventing reproductive dysfunction associated with ovarian aging. Second, gonadotropin ovarian stimulation can influence gene expression in mouse ovaries (Marshall & Rivera, 2018). Older women undergoing IVF respond poorly to ovarian stimulation protocols, and their oocytes are often of lower yield and quality compared with those from younger women (Conforti et al., 2019; Lee et al., 2012). Transcriptomic analysis of gonadotropin‐stimulated ovaries can provide insight into the molecular processes contributing to poor response to gonadotropin stimulation for aged women receiving IVF. Third, the ovaries were removed immediately after ovulation at the time of analysis in this study, and hence, they were in the luteal phase. Therefore, they could not have been responsible for folliculogenesis and oocyte maturation. Over the reproductive lifespan of a female, the number of follicles and oocytes decreases (Llibertos et al., 2021). It is thought that there was a difference in the expression of folliculogenesis‐related genes due to the age‐related decrease in the number of follicles.

In summary, we determined age‐related changes in the genetic regulation of ovarian function. Integrative analysis showed that ovarian aging can alter miRNA‐mediated epigenetic modifications involved in the regulation of folliculogenesis, oocyte growth, and steroidogenesis, leading to decreased oocyte quality.

## CONFLICT OF INTEREST

Authors declare no conflict of interest for this article.

## Supporting information


**Table S1.** List of genes upregulated in old compared to young mice

**Table S2.** List of genes downregulated in old compared to young mice

## Data Availability

The datasets generated and/or analyzed during the current study are available from the corresponding author upon reasonable request. RNA seq and small RNA seq data have been registered in the National Center for Biotechnology Information (https://www.ncbi.nlm.nih.gov/Traces/study/?acc=PRJNA760963).
