# Supplementary material for: Comprehensive analysis of miRNA–mRNA interactions in ovaries of aged mice
Source: Anim Sci J. 2022 Apr 13;93(1):e13721. doi: 10.1111/asj.13721 (PMC9285582; doi:10.1111/asj.13721)
Supplement: Supplementary file 1 — Table S1. List of genes upregulated in old compared to young mice [file ASJ-93-0-s001.docx]

**Supplementary Table 1. List of genes upregulated in old compared to young mice**

| Gene symbol | Gene ID | Fold change | p-value |
| --- | --- | --- | --- |
| Plvap | plasmalemma vesicle associated protein | 2.023 | <0.001 |
| Plekha6 | pleckstrin homology domain containing, family A member 6 | 2.042 | <0.001 |
| Runx3 | runt related transcription factor 3 | 2.043 | <0.001 |
| Ptpru | protein tyrosine phosphatase, receptor type, U | 2.056 | 0.006 |
| Cyp4f14 | cytochrome P450, family 4, subfamily f, polypeptide 14 | 2.058 | <0.001 |
| Ifit3 | interferon-induced protein with tetratricopeptide repeats 3 | 2.066 | 0.010 |
| C3 | complement component 3 | 2.068 | <0.001 |
| Apol9b | apolipoprotein L 9b | 2.082 | 0.010 |
| H2-K2 | histocompatibility 2, K region locus 2 | 2.087 | 0.001 |
| Plin5 | perilipin 5 | 2.089 | <0.001 |
| Mgst2 | microsomal glutathione S-transferase 2 | 2.099 | <0.001 |
| Oas1a | 2'-5' oligoadenylate synthetase 1A | 2.102 | 0.004 |
| Gm10789 | predicted gene 10789 | 2.127 | 0.001 |
| Cp | ceruloplasmin | 2.155 | <0.001 |
| Slc35f2 | solute carrier family 35, member F2 | 2.192 | <0.001 |
| Kcnd2 | potassium voltage-gated channel, Shal-related family, member 2 | 2.231 | 0.001 |
| Adgrg7 | adhesion G protein-coupled receptor G7 | 2.247 | 0.005 |
| Homer2 | homer scaffolding protein 2 | 2.272 | <0.001 |
| Ces2e | carboxylesterase 2E | 2.273 | 0.003 |
| Gabrb1 | gamma-aminobutyric acid (GABA) A receptor, subunit beta 1 | 2.300 | 0.001 |
| Serpina3g | serine (or cysteine) peptidase inhibitor, clade A, member 3G | 2.336 | <0.001 |
| Slpi | secretory leukocyte peptidase inhibitor | 2.369 | 0.001 |
| Rhbdl3 | rhomboid, veinlet-like 3 (Drosophila) | 2.374 | <0.001 |
| Abcb1b | ATP-binding cassette, sub-family B (MDR/TAP), member 1B | 2.391 | <0.001 |
| 4930486L24Rik | RIKEN cDNA 4930486L24 gene | 2.399 | <0.001 |
| Tgtp2 | T cell specific GTPase 2 | 2.402 | 0.006 |
| Fam19a2 | family with sequence similarity 19, member A2 | 2.418 | <0.001 |
| Gpihbp1 | GPI-anchored HDL-binding protein 1 | 2.465 | <0.001 |
| Radil | Ras association and DIL domains | 2.470 | <0.001 |
| Wfdc17 | WAP four-disulfide core domain 17 | 2.520 | <0.001 |
| Hsd3b6 | hydroxy-delta-5-steroid dehydrogenase, 3 beta- and steroid delta-isomerase 6 | 2.532 | 0.001 |
| Atp12a | ATPase, H+/K+ transporting, nongastric, alpha polypeptide | 2.533 | <0.001 |
| Rgn | regucalcin | 2.583 | 0.005 |
| H2-Q9 | histocompatibility 2, Q region locus 9 | 2.586 | <0.001 |
| Itgb7 | integrin beta 7 | 2.605 | 0.005 |
| Pirb | paired Ig-like receptor B | 2.619 | 0.002 |
| F2rl3 | coagulation factor II (thrombin) receptor-like 3 | 2.637 | <0.001 |
| Plet1 | placenta expressed transcript 1 | 2.652 | 0.001 |
| Acan | aggrecan | 2.660 | 0.002 |
| Gm4951 | predicted gene 4951 | 2.685 | <0.001 |
| Rsad2 | radical S-adenosyl methionine domain containing 2 | 2.715 | <0.001 |
| H2-DMb1 | histocompatibility 2, class II, locus Mb1 | 2.750 | 0.001 |
| Gimap3 | GTPase, IMAP family member 3 | 2.785 | 0.003 |
| Bpifc | BPI fold containing family C | 2.834 | 0.002 |
| Pla2g2d | phospholipase A2, group IID | 2.892 | 0.005 |
| AW112010 | expressed sequence AW112010 | 2.908 | 0.001 |
| Atp6v0d2 | ATPase, H+ transporting, lysosomal V0 subunit D2 | 2.998 | 0.004 |
| Snhg11 | small nucleolar RNA host gene 11 | 3.113 | 0.001 |
| Mmp12 | matrix metallopeptidase 12 | 3.132 | 0.005 |
| Nfasc | neurofascin | 3.186 | 0.005 |
| Cyp26b1 | cytochrome P450, family 26, subfamily b, polypeptide 1 | 3.192 | <0.001 |
| Il7r | interleukin 7 receptor | 3.219 | 0.009 |
| Lix1 | limb and CNS expressed 1 | 3.255 | <0.001 |
| Slco1a4 | solute carrier organic anion transporter family, member 1a4 | 3.267 | 0.002 |
| Ankrd55 | ankyrin repeat domain 55 | 3.372 | <0.001 |
| Arhgap15 | Rho GTPase activating protein 15 | 3.430 | <0.001 |
| BC051142 | cDNA sequence BC051142 | 3.431 | 0.001 |
| Il33 | interleukin 33 | 3.510 | <0.001 |
| Clec4d | C-type lectin domain family 4, member d | 3.617 | 0.007 |
| Tmem178 | transmembrane protein 178 | 3.649 | <0.001 |
| Gm13003 | predicted gene 13003 | 3.663 | <0.001 |
| Sectm1a | secreted and transmembrane 1A | 3.695 | <0.001 |
| Cdkn2a | cyclin-dependent kinase inhibitor 2A | 3.860 | 0.001 |
| Cxcr6 | chemokine (C-X-C motif) receptor 6 | 3.947 | 0.001 |
| Mal | myelin and lymphocyte protein, T cell differentiation protein | 4.005 | <0.001 |
| Naip6 | NLR family, apoptosis inhibitory protein 6 | 4.222 | <0.001 |
| Adcyap1 | adenylate cyclase activating polypeptide 1 | 4.745 | <0.001 |
| Cpxm2 | carboxypeptidase X 2 (M14 family) | 5.541 | <0.001 |
| Esm1 | endothelial cell-specific molecule 1 | 5.624 | <0.001 |
| Fabp4 | fatty acid binding protein 4, adipocyte | 6.108 | <0.001 |
| Gpnmb | glycoprotein (transmembrane) nmb | 6.329 | 0.001 |

Comparison of expression data from young and old mice revealed 71 differentially upregulated genes with fold changes > 2.0, and *p* < 0.01. Young: 12-week-old mice; old: 44-week-old mice.
