# Supplementary material for: Comprehensive analysis of miRNA–mRNA interactions in ovaries of aged mice
Source: Anim Sci J. 2022 Apr 13;93(1):e13721. doi: 10.1111/asj.13721 (PMC9285582; doi:10.1111/asj.13721)
Supplement: Supplementary file 2 — Table S2. List of genes downregulated in old compared to young mice [file ASJ-93-0-s002.docx]

**Supplementary Table 2. List of genes downregulated in old compared to young mice**

| Gene symbol | Gene ID | Fold change | p-value |
| --- | --- | --- | --- |
| Rnase1 | ribonuclease, RNase A family, 1 (pancreatic) | 0.141 | <0.001 |
| Col9a2 | collagen, type IX, alpha 2 | 0.163 | <0.001 |
| Gulo | gulonolactone (L-) oxidase | 0.169 | <0.001 |
| Apoa4 | apolipoprotein A-IV | 0.175 | <0.001 |
| Nmu | neuromedin U | 0.179 | 0.006 |
| Arhgap20os | Rho GTPase activating protein 20, opposite strand | 0.182 | <0.001 |
| Zar1l | zygote arrest 1-like | 0.197 | <0.001 |
| Trim61 | tripartite motif-containing 61 | 0.202 | <0.001 |
| 5330411J11Rik | RIKEN cDNA 5330411J11 gene | 0.212 | <0.001 |
| Crabp2 | cellular retinoic acid binding protein II | 0.216 | <0.001 |
| Oas1h | 2'-5' oligoadenylate synthetase 1H | 0.217 | <0.001 |
| Kcnq5 | potassium voltage-gated channel, subfamily Q, member 5 | 0.217 | 0.005 |
| Lect1 | leukocyte cell derived chemotaxin 1 | 0.219 | <0.001 |
| Gm2694 | predicted gene 2694 | 0.220 | <0.001 |
| Sel1l3 | sel-1 suppressor of lin-12-like 3 (C. elegans) | 0.221 | <0.001 |
| Lnx1 | ligand of numb-protein X 1 | 0.223 | 0.001 |
| Rfpl4 | ret finger protein-like 4 | 0.227 | <0.001 |
| Fbxw20 | F-box and WD-40 domain protein 20 | 0.237 | <0.001 |
| 4930562C15Rik | RIKEN cDNA 4930562C15 gene | 0.239 | <0.001 |
| Mos | Moloney sarcoma oncogene | 0.240 | <0.001 |
| Tdrd1 | tudor domain containing 1 | 0.241 | 0.001 |
| Crtac1 | cartilage acidic protein 1 | 0.243 | 0.001 |
| C87977 | expressed sequence C87977 | 0.249 | 0.001 |
| Nlrp9b | NLR family, pyrin domain containing 9B | 0.250 | <0.001 |
| Apoa1 | apolipoprotein A-I | 0.251 | <0.001 |
| Gm4961 | predicted pseudogene 4961 | 0.254 | <0.001 |
| Zp2 | zona pellucida glycoprotein 2 | 0.256 | <0.001 |
| Nlrp14 | NLR family, pyrin domain containing 14 | 0.257 | <0.001 |
| Oog4 | oogenesin 4 | 0.258 | <0.001 |
| Gm813 | predicted gene 813 | 0.258 | <0.001 |
| C87499 | expressed sequence C87499 | 0.258 | 0.002 |
| Gm9961 | predicted gene 9961 | 0.259 | <0.001 |
| Nobox | NOBOX oogenesis homeobox | 0.260 | <0.001 |
| Khdc3 | KH domain containing 3, subcortical maternal complex member | 0.260 | <0.001 |
| Oas1e | 2'-5' oligoadenylate synthetase 1E | 0.261 | <0.001 |
| Mycl | v-myc avian myelocytomatosis viral oncogene lung carcinoma derived | 0.261 | <0.001 |
| Tcl1 | T cell lymphoma breakpoint 1 | 0.261 | <0.001 |
| Kpna7 | karyopherin alpha 7 (importin alpha 8) | 0.262 | <0.001 |
| Gdap1 | ganglioside-induced differentiation-associated-protein 1 | 0.263 | <0.001 |
| Mbl2 | mannose-binding lectin (protein C) 2 | 0.264 | 0.001 |
| Gtsf1 | gametocyte specific factor 1 | 0.266 | 0.001 |
| Oosp3 | oocyte secreted protein 3 | 0.266 | 0.001 |
| Speer5-ps1 | spermatogenesis associated glutamate (E)-rich protein 5, pseudogene 1 | 0.268 | <0.001 |
| AU015836 | expressed sequence AU015836 | 0.269 | <0.001 |
| Smc1b | structural maintenance of chromosomes 1B | 0.271 | <0.001 |
| E330021D16Rik | RIKEN cDNA E330021D16 gene | 0.271 | <0.001 |
| Eif4e1b | eukaryotic translation initiation factor 4E family member 1B | 0.271 | <0.001 |
| D6Ertd527e | DNA segment, Chr 6, ERATO Doi 527, expressed | 0.271 | <0.001 |
| Zfp385b | zinc finger protein 385B | 0.272 | 0.001 |
| Nnat | neuronatin | 0.274 | 0.001 |
| Zar1 | zygote arrest 1 | 0.274 | <0.001 |
| Zp1 | zona pellucida glycoprotein 1 | 0.274 | <0.001 |
| Slc18a2 | solute carrier family 18 (vesicular monoamine), member 2 | 0.277 | <0.001 |
| Pln | phospholamban | 0.280 | <0.001 |
| Zp3 | zona pellucida glycoprotein 3 | 0.281 | <0.001 |
| E330034G19Rik | RIKEN cDNA E330034G19 gene | 0.281 | <0.001 |
| C87414 | expressed sequence C87414 | 0.282 | <0.001 |
| Ccno | cyclin O | 0.282 | <0.001 |
| Esr2 | estrogen receptor 2 (beta) | 0.285 | <0.001 |
| Gm10436 | predicted gene 10436 | 0.286 | <0.001 |
| H1foo | H1 histone family, member O, oocyte-specific | 0.286 | <0.001 |
| Omt2a | oocyte maturation, alpha | 0.287 | <0.001 |
| Bmp15 | bone morphogenetic protein 15 | 0.288 | <0.001 |
| C86187 | expressed sequence C86187 | 0.288 | <0.001 |
| Gm2042 | predicted gene 2042 | 0.288 | <0.001 |
| Oas1d | 2'-5' oligoadenylate synthetase 1D | 0.289 | <0.001 |
| Gm13023 | predicted gene 13023 | 0.289 | <0.001 |
| Cpa1 | carboxypeptidase A1, pancreatic | 0.290 | <0.001 |
| Apoc3 | apolipoprotein C-III | 0.290 | <0.001 |
| Ccdc158 | coiled-coil domain containing 158 | 0.292 | 0.003 |
| Shbg | sex hormone binding globulin | 0.293 | <0.001 |
| Astl | astacin-like metalloendopeptidase (M12 family) | 0.294 | <0.001 |
| Fbxw19 | F-box and WD-40 domain protein 19 | 0.294 | 0.001 |
| Tcl1b1 | T cell leukemia/lymphoma 1B, 1 | 0.294 | <0.001 |
| Khdc1b | KH domain containing 1B | 0.294 | <0.001 |
| Gm5434 | predicted gene 5434 | 0.296 | 0.001 |
| Unc13c | unc-13 homolog C (C. elegans) | 0.297 | <0.001 |
| Nlrp4a | NLR family, pyrin domain containing 4A | 0.297 | 0.004 |
| Oas1c | 2'-5' oligoadenylate synthetase 1C | 0.298 | 0.001 |
| Izumo1r | IZUMO1 receptor, JUNO | 0.299 | <0.001 |
| Gm15698 | predicted gene 15698 | 0.299 | <0.001 |
| Fbxw24 | F-box and WD-40 domain protein 24 | 0.301 | <0.001 |
| Wfdc10 | WAP four-disulfide core domain 10 | 0.301 | <0.001 |
| Fbxw27 | F-box and WD-40 domain protein 27 | 0.303 | <0.001 |
| Obox5 | oocyte specific homeobox 5 | 0.303 | 0.001 |
| Zdbf2 | zinc finger, DBF-type containing 2 | 0.304 | <0.001 |
| Ooep | oocyte expressed protein | 0.305 | <0.001 |
| Cbs | cystathionine beta-synthase | 0.307 | <0.001 |
| Pramef12 | PRAME family member 12 | 0.309 | <0.001 |
| Fbxw14 | F-box and WD-40 domain protein 14 | 0.310 | <0.001 |
| Dkkl1 | dickkopf-like 1 | 0.310 | 0.004 |
| E330017A01Rik | RIKEN cDNA E330017A01 gene | 0.311 | <0.001 |
| Fbxw16 | F-box and WD-40 domain protein 16 | 0.311 | <0.001 |
| Arhgef28 | Rho guanine nucleotide exchange factor (GEF) 28 | 0.312 | <0.001 |
| Nlrp2 | NLR family, pyrin domain containing 2 | 0.314 | <0.001 |
| AU022751 | expressed sequence AU022751 | 0.315 | <0.001 |
| Gm1965 | predicted gene 1965 | 0.316 | <0.001 |
| Rspo2 | R-spondin 2 | 0.316 | 0.001 |
| Unc5c | unc-5 netrin receptor C | 0.316 | <0.001 |
| Rbm20 | RNA binding motif protein 20 | 0.318 | 0.001 |
| Dnah10 | dynein, axonemal, heavy chain 10 | 0.320 | 0.001 |
| Ntrk2 | neurotrophic tyrosine kinase, receptor, type 2 | 0.320 | <0.001 |
| Tcl1b4 | T cell leukemia/lymphoma 1B, 4 | 0.320 | 0.001 |
| Serpina3a | serine (or cysteine) peptidase inhibitor, clade A, member 3A | 0.320 | <0.001 |
| Nlrp4f | NLR family, pyrin domain containing 4F | 0.321 | <0.001 |
| Jak3 | Janus kinase 3 | 0.322 | <0.001 |
| Slc38a5 | solute carrier family 38, member 5 | 0.322 | 0.005 |
| Khdc1a | KH domain containing 1A | 0.323 | <0.001 |
| Ano4 | anoctamin 4 | 0.324 | <0.001 |
| Derl3 | Der1-like domain family, member 3 | 0.324 | <0.001 |
| Rhd | Rh blood group, D antigen | 0.326 | <0.001 |
| Pabpn1l | poly(A)binding protein nuclear 1-like | 0.328 | <0.001 |
| Bmpr1b | bone morphogenetic protein receptor, type 1B | 0.328 | 0.001 |
| Gdf9 | growth differentiation factor 9 | 0.329 | <0.001 |
| Sohlh1 | spermatogenesis and oogenesis specific basic helix-loop-helix 1 | 0.329 | 0.002 |
| Itih2 | inter-alpha trypsin inhibitor, heavy chain 2 | 0.330 | <0.001 |
| Tex19.1 | testis expressed gene 19.1 | 0.330 | <0.001 |
| Fbxw18 | F-box and WD-40 domain protein 18 | 0.330 | <0.001 |
| Klf17 | Kruppel-like factor 17 | 0.331 | 0.001 |
| Agt | angiotensinogen (serpin peptidase inhibitor, clade A, member 8) | 0.331 | 0.001 |
| Dppa5a | developmental pluripotency associated 5A | 0.331 | <0.001 |
| Ntng1 | netrin G1 | 0.331 | 0.001 |
| Omt2b | oocyte maturation, beta | 0.334 | <0.001 |
| Gm13103 | predicted gene 13103 | 0.334 | 0.001 |
| AI427809 | expressed sequence AI427809 | 0.334 | <0.001 |
| Dazl | deleted in azoospermia-like | 0.334 | 0.002 |
| Padi6 | peptidyl arginine deiminase, type VI | 0.336 | <0.001 |
| Tdrd5 | tudor domain containing 5 | 0.340 | <0.001 |
| Xkr5 | X-linked Kx blood group related 5 | 0.341 | 0.002 |
| Ddx4 | DEAD (Asp-Glu-Ala-Asp) box polypeptide 4 | 0.341 | 0.001 |
| Nlrp4b | NLR family, pyrin domain containing 4B | 0.342 | 0.005 |
| Umodl1 | uromodulin-like 1 | 0.343 | 0.001 |
| Btg4 | B cell translocation gene 4 | 0.343 | <0.001 |
| Obox1 | oocyte specific homeobox 1 | 0.344 | <0.001 |
| Slc30a3 | solute carrier family 30 (zinc transporter), member 3 | 0.345 | <0.001 |
| Grm4 | glutamate receptor, metabotropic 4 | 0.345 | 0.002 |
| Tmem52 | transmembrane protein 52 | 0.347 | 0.004 |
| Cables1 | CDK5 and Abl enzyme substrate 1 | 0.348 | <0.001 |
| Obox2 | oocyte specific homeobox 2 | 0.349 | <0.001 |
| Fbxw15 | F-box and WD-40 domain protein 15 | 0.349 | <0.001 |
| Npm2 | nucleophosmin/nucleoplasmin 2 | 0.350 | <0.001 |
| Bcl2l10 | Bcl2-like 10 | 0.352 | <0.001 |
| Esrp1 | epithelial splicing regulatory protein 1 | 0.353 | <0.001 |
| Gyltl1b | glycosyltransferase-like 1B | 0.353 | <0.001 |
| Oosp1 | oocyte secreted protein 1 | 0.355 | 0.001 |
| Fcrls | Fc receptor-like S, scavenger receptor | 0.355 | <0.001 |
| Fbxw28 | F-box and WD-40 domain protein 28 | 0.356 | 0.001 |
| Fhit | fragile histidine triad gene | 0.356 | <0.001 |
| Vrtn | vertebrae development associated | 0.356 | 0.003 |
| Fbxw21 | F-box and WD-40 domain protein 21 | 0.356 | <0.001 |
| Sall4 | sal-like 4 (Drosophila) | 0.358 | <0.001 |
| Ank3 | ankyrin 3, epithelial | 0.358 | <0.001 |
| Dppa3 | developmental pluripotency-associated 3 | 0.359 | <0.001 |
| Ninj2 | ninjurin 2 | 0.360 | 0.002 |
| Cpsf4l | cleavage and polyadenylation specific factor 4-like | 0.360 | <0.001 |
| 4933427D06Rik | RIKEN cDNA 4933427D06 gene | 0.364 | <0.001 |
| Fbxw22 | F-box and WD-40 domain protein 22 | 0.366 | 0.001 |
| Styk1 | serine/threonine/tyrosine kinase 1 | 0.366 | 0.006 |
| Trim60 | tripartite motif-containing 60 | 0.368 | <0.001 |
| Col11a1 | collagen, type XI, alpha 1 | 0.371 | <0.001 |
| Dpt | dermatopontin | 0.373 | 0.005 |
| Tex15 | testis expressed gene 15 | 0.374 | <0.001 |
| Angptl1 | angiopoietin-like 1 | 0.374 | <0.001 |
| Hpgd | hydroxyprostaglandin dehydrogenase 15 (NAD) | 0.378 | <0.001 |
| Ybx2 | Y box protein 2 | 0.378 | <0.001 |
| Ifit1bl2 | interferon induced protein with tetratricopeptide repeats 1B like 2 | 0.379 | <0.001 |
| Tceal6 | transcription elongation factor A (SII)-like 6 | 0.380 | 0.009 |
| Fbxw26 | F-box and WD-40 domain protein 26 | 0.386 | <0.001 |
| Fgd3 | FYVE, RhoGEF and PH domain containing 3 | 0.387 | <0.001 |
| Igf2bp2 | insulin-like growth factor 2 mRNA binding protein 2 | 0.387 | <0.001 |
| Il13ra2 | interleukin 13 receptor, alpha 2 | 0.387 | 0.003 |
| Fgf8 | fibroblast growth factor 8 | 0.389 | <0.001 |
| Oog1 | oogenesin 1 | 0.390 | 0.001 |
| Slc6a4 | solute carrier family 6 (neurotransmitter transporter, serotonin), member 4 | 0.397 | 0.002 |
| Elavl3 | ELAV (embryonic lethal, abnormal vision, Drosophila)-like 3 (Hu antigen C) | 0.397 | 0.001 |
| Nlrp5 | NLR family, pyrin domain containing 5 | 0.400 | <0.001 |
| Gm12295 | predicted gene 12295 | 0.401 | 0.008 |
| Bcan | brevican | 0.402 | 0.001 |
| Oog3 | oogenesin 3 | 0.404 | 0.004 |
| Gm13084 | predicted gene 13084 | 0.405 | 0.002 |
| Wee2 | WEE1 homolog 2 (S. pombe) | 0.405 | <0.001 |
| Tcl1b2 | T cell leukemia/lymphoma 1B, 2 | 0.406 | 0.002 |
| Tfap2e | transcription factor AP-2, epsilon | 0.406 | <0.001 |
| Raet1b | Retinoic acid early-inducible protein 1-beta | 0.407 | <0.001 |
| Kctd14 | potassium channel tetramerisation domain containing 14 | 0.408 | <0.001 |
| Nphs2 | nephrosis 2, podocin | 0.409 | 0.001 |
| Kcnj3 | potassium inwardly-rectifying channel, subfamily J, member 3 | 0.411 | <0.001 |
| Lrrc10b | leucine rich repeat containing 10B | 0.412 | <0.001 |
| Gdpd2 | glycerophosphodiester phosphodiesterase domain containing 2 | 0.412 | <0.001 |
| E330011O21Rik | RIKEN cDNA E330011O21 gene | 0.414 | 0.005 |
| Defb19 | defensin beta 19 | 0.417 | <0.001 |
| Pdlim3 | PDZ and LIM domain 3 | 0.419 | 0.006 |
| Dpysl5 | dihydropyrimidinase-like 5 | 0.419 | 0.003 |
| Uchl1 | ubiquitin carboxy-terminal hydrolase L1 | 0.421 | <0.001 |
| Tnni3 | troponin I, cardiac 3 | 0.421 | <0.001 |
| Alpk3 | alpha-kinase 3 | 0.422 | 0.001 |
| Fam46b | family with sequence similarity 46, member B | 0.422 | 0.001 |
| Pla2g4c | phospholipase A2, group IVC (cytosolic, calcium-independent) | 0.422 | <0.001 |
| Zbtb16 | zinc finger and BTB domain containing 16 | 0.424 | 0.006 |
| Perp | PERP, TP53 apoptosis effector | 0.424 | <0.001 |
| Omd | osteomodulin | 0.425 | 0.002 |
| Ccdc3 | coiled-coil domain containing 3 | 0.426 | <0.001 |
| Foxp2 | forkhead box P2 | 0.429 | <0.001 |
| Car14 | carbonic anhydrase 14 | 0.429 | 0.001 |
| Rgs2 | regulator of G-protein signaling 2 | 0.430 | <0.001 |
| Csmd1 | CUB and Sushi multiple domains 1 | 0.432 | 0.009 |
| Aff3 | AF4/FMR2 family, member 3 | 0.434 | <0.001 |
| Myo5c | myosin VC | 0.435 | 0.001 |
| Slc6a15 | solute carrier family 6 (neurotransmitter transporter), member 15 | 0.435 | <0.001 |
| Rimkla | ribosomal modification protein rimK-like family member A | 0.436 | 0.003 |
| Abca1 | ATP-binding cassette, sub-family A (ABC1), member 1 | 0.437 | <0.001 |
| Elovl2 | elongation of very long chain fatty acids (FEN1/Elo2, SUR4/Elo3, yeast)-like 2 | 0.437 | 0.001 |
| Mtfr2 | mitochondrial fission regulator 2 | 0.437 | <0.001 |
| Sytl4 | synaptotagmin-like 4 | 0.438 | <0.001 |
| Bex4 | brain expressed X-linked 4 | 0.438 | <0.001 |
| Myocd | myocardin | 0.438 | <0.001 |
| Cbr2 | carbonyl reductase 2 | 0.440 | 0.001 |
| Dmkn | dermokine | 0.440 | 0.008 |
| Rhpn2 | rhophilin, Rho GTPase binding protein 2 | 0.441 | <0.001 |
| 4930480K15Rik | RIKEN cDNA 4930480K15 gene | 0.442 | 0.006 |
| Dhh | desert hedgehog | 0.443 | <0.001 |
| Nrxn1 | neurexin I | 0.444 | <0.001 |
| Creb3l3 | cAMP responsive element binding protein 3-like 3 | 0.444 | 0.001 |
| Dtna | dystrobrevin alpha | 0.445 | <0.001 |
| Nos2 | nitric oxide synthase 2, inducible | 0.445 | 0.001 |
| Mybpc3 | myosin binding protein C, cardiac | 0.446 | 0.006 |
| Fndc5 | fibronectin type III domain containing 5 | 0.446 | <0.001 |
| Mns1 | meiosis-specific nuclear structural protein 1 | 0.447 | <0.001 |
| Nipal1 | NIPA-like domain containing 1 | 0.452 | <0.001 |
| B3galt2 | UDP-Gal:betaGlcNAc beta 1,3-galactosyltransferase, polypeptide 2 | 0.452 | <0.001 |
| Ihh | Indian hedgehog | 0.456 | <0.001 |
| Fam78a | family with sequence similarity 78, member A | 0.457 | 0.001 |
| Elavl2 | ELAV (embryonic lethal, abnormal vision, Drosophila)-like 2 (Hu antigen B) | 0.459 | <0.001 |
| Hsd11b2 | hydroxysteroid 11-beta dehydrogenase 2 | 0.460 | <0.001 |
| Dbndd1 | dysbindin (dystrobrevin binding protein 1) domain containing 1 | 0.460 | <0.001 |
| Arxes2 | adipocyte-related X-chromosome expressed sequence 2 | 0.460 | <0.001 |
| Scube2 | signal peptide, CUB domain, EGF-like 2 | 0.462 | <0.001 |
| Msln | mesothelin | 0.465 | <0.001 |
| Pcdh11x | protocadherin 11 X-linked | 0.467 | 0.003 |
| Syt7 | synaptotagmin VII | 0.467 | <0.001 |
| Reln | reelin | 0.470 | 0.001 |
| Rbfox1 | RNA binding protein, fox-1 homolog (C. elegans) 1 | 0.471 | 0.001 |
| Cd164l2 | CD164 sialomucin-like 2 | 0.472 | <0.001 |
| Pcsk6 | proprotein convertase subtilisin/kexin type 6 | 0.472 | <0.001 |
| Aard | alanine and arginine rich domain containing protein | 0.472 | 0.009 |
| Dsp | desmoplakin | 0.473 | <0.001 |
| Slc38a4 | solute carrier family 38, member 4 | 0.477 | 0.007 |
| Bend4 | BEN domain containing 4 | 0.478 | 0.005 |
| Zfp940 | zinc finger protein 940 | 0.479 | 0.009 |
| Bcl11a | B cell CLL/lymphoma 11A (zinc finger protein) | 0.479 | <0.001 |
| Susd3 | sushi domain containing 3 | 0.480 | <0.001 |
| Lrrc16b | leucine rich repeat containing 16B | 0.480 | 0.008 |
| Gm1987 | predicted gene 1987 | 0.480 | 0.004 |
| Ccl21a | chemokine (C-C motif) ligand 21A (serine) | 0.480 | 0.005 |
| Cdo1 | cysteine dioxygenase 1, cytosolic | 0.482 | 0.001 |
| Bmp3 | bone morphogenetic protein 3 | 0.483 | 0.010 |
| Slc45a3 | solute carrier family 45, member 3 | 0.484 | <0.001 |
| Fmn2 | formin 2 | 0.484 | 0.008 |
| 4930550C14Rik | RIKEN cDNA 4930550C14 gene | 0.484 | 0.001 |
| Kazald1 | Kazal-type serine peptidase inhibitor domain 1 | 0.485 | 0.003 |
| Rapgefl1 | Rap guanine nucleotide exchange factor (GEF)-like 1 | 0.485 | <0.001 |
| 1500015O10Rik | RIKEN cDNA 1500015O10 gene | 0.486 | 0.005 |
| Batf3 | basic leucine zipper transcription factor, ATF-like 3 | 0.488 | 0.009 |
| Myoc | myocilin | 0.489 | <0.001 |
| Hunk | hormonally upregulated Neu-associated kinase | 0.489 | 0.003 |
| Adgrg1 | adhesion G protein-coupled receptor G1 | 0.491 | <0.001 |
| Tubb2b | tubulin, beta 2B class IIB | 0.491 | <0.001 |
| Metrnl | meteorin, glial cell differentiation regulator-like | 0.491 | <0.001 |
| Me3 | malic enzyme 3, NADP(+)-dependent, mitochondrial | 0.493 | <0.001 |
| F13a1 | coagulation factor XIII, A1 subunit | 0.494 | 0.005 |
| Snora47 | small nucleolar RNA, H/ACA box 47 | 0.499 | 0.008 |
| Pcyt1b | phosphate cytidylyltransferase 1, choline, beta isoform | 0.499 | 0.004 |

Comparison of expression data from young and old mice revealed 272 differentially downregulated genes with fold changes > 2.0, and *p* < 0.01. Young: 12-week-old mice; old: 44-week-old mice.
